# Exploring the direct and indirect impacts of climate variability on armed conflict in South Asia

**DOI:** 10.1016/j.isci.2022.105258

**Published:** 2022-10-19

**Authors:** Xiaolan Xie, Mengmeng Hao, Fangyu Ding, David Helman, Jürgen Scheffran, Qian Wang, Quansheng Ge, Dong Jiang

**Affiliations:** 1 State Key Laboratory of Resources and Environmental Information System, Institute of Geographic Sciences and Natural Resources Research, Chinese Academy of Sciences, Beijing 100101, China; 2 College of Resources and Environment, University of Chinese Academy of Sciences, Beijing 100049, China; 3 Key Laboratory of Carrying Capacity Assessment for Resource and Environment, Ministry of Land & Resources, Beijing 100101, China; 4 Institute of Environmental Sciences (Soil & Water), The Robert H. Smith Faculty of Agriculture, Food and Environment, The Hebrew University, Rehovot 7610001, Israel; 5 Advanced School for Environmental Studies, The Hebrew University of Jerusalem, Jerusalem 91905, Israel; 6 Institute of Geography, Center for Earth System Research and Sustainability, University of Hamburg, Hamburg 20144, Germany; 7 Centre for Tropical Medicine, Nuffield Department of Clinical Medicine, University of Oxford, Oxford OX13QR, UK

**Keywords:** Global change, Environmental event, Political science, Sociology

## Abstract

Although numerous studies have examined the effects of climate variability on armed conflict, the complexity of these linkages requires deeper understanding to assess the causes and effects. Here, we assembled an extensive database of armed conflict, climate, and non-climate data for South Asia. We used structural equation modeling to quantify both the direct and indirect impacts of climate variability on armed conflict. We found that precipitation impacts armed conflict via direct and indirect effects which are contradictory in sign. Temperature affects armed conflict only through a direct path, while indirect effects were insignificant. Yet, an in-depth analysis of indirect effects showed that the net impact is weak due to two strong contradictory effects offsetting each other. Our findings illustrate the complex link between climate variability and armed conflict, highlighting the importance of a detailed analysis of South Asia’s underlying mechanisms at the regional scale.

## Highlights

• Using SEM, we explore how climate variability affects armed conflict in South Asia
• Climate variability has both complex direct and indirect impacts on armed conflict
• The indirect effects of climate variability on armed conflict offset each other
• Emphasizing the value of the detailed division of climate-conflict mechanisms

## Graphical abstract

**Figure:**
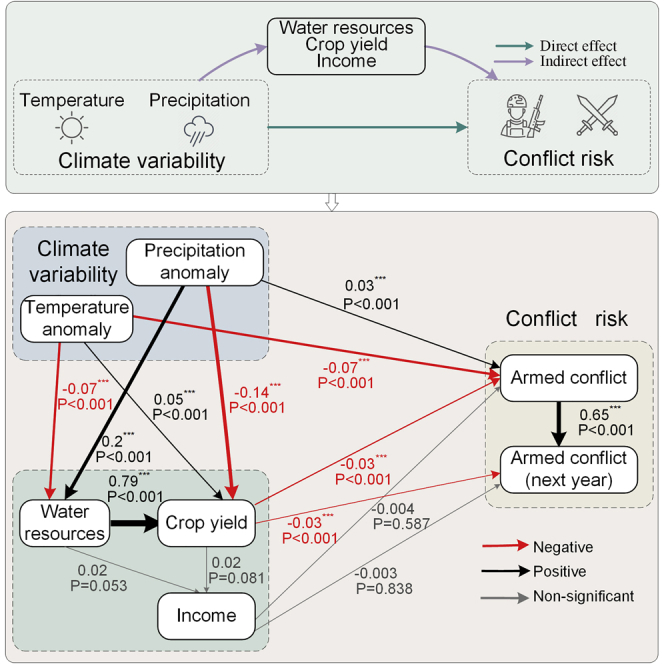

## Introduction

Armed conflict has remained one of the greatest threats to human security (Schleussner et al., 2016). Determining the underlying processes and driving factors of armed conflict has been an active area of scientific research, and an in-depth understanding of the drivers of armed conflict may help to prevent armed conflict (Hsiang et al., 2013; Lim et al., 2007). There is increasing evidence that climate variability may have devastating effects on human living conditions by driving risks to livelihoods and incomes, compounded by forced displacement, food price shocks, and land-use disputes (Diffenbaugh and Burke, 2019; Lobell et al., 2011; Stenzel et al., 2021; von Uexkull and Buhaug, 2021; Wheeler and von Braun, 2013). These findings encourage the suggestion that climate variability may have impacts on armed conflict (Burke et al., 2009; O'Loughlin et al., 2012).

During the last 10 to 15 years, researchers have conducted numerous studies to examine how climate variability, often represented by temperature and precipitation, possibly affects armed conflict (Hsiang et al., 2013; Jun and Sethi, 2021; O'Loughlin et al., 2014; Roche et al., 2020; Salehyan and Hendrix, 2014). Previous research proposed potential direct and indirect pathways linking climate variability with armed conflict. It has been suggested that climate has a direct effect on armed conflict via physiological and/or psychological factors. For example, based on laboratory research, Anderson (1989) showed that uncomfortable temperatures increase negative effects that lead to personal aggression and violence.

Besides affecting armed conflict directly, some studies suggest that climate variability influences conflict indirectly through economic linkages (Koubi et al., 2012; Roche et al., 2020; Zhang et al., 2007). The hypothesis is that some conflict is led by climate-depressed economic output. For example, climate variability could induce a drop in agricultural incomes, which would trigger the onset of conflict, especially in agricultural-dependent regions. The reduced nation incomes resulting from the climate crisis would affect the duration and intensity of the conflict (Koubi, 2019). Zhang et al. (2007) and Jun and Sethi (2021), which used historical data to analyze the outbreak of violent conflict over several centuries, showed an increasing risk of conflict when agricultural production and food shortages occur in agrarian societies. As de Mesquita and Smith (2017) suggested, when the capacity of leaders in a country is curtailed by climate-driven resource shortage, there will be opportunities for their opponents to organize political resistance to overthrow them from power. Schilling et al. (2020) found that the adverse effect of climate variability on food supply had aggravated broadly based social unrest such as the well-known “Arab Spring”. Generally, the above studies suggest that climate variability might indirectly affect conflict through the economy.

Although empirical literature found an impact of climate variability on armed conflict and developed some clear ideas of how climate variability might affect armed conflict (Koubi, 2019), there remains a void in the quantification of the direct and indirect impacts of climate variability on armed conflict in certain regions. At the same time, the impact of climate variability on armed conflict is usually studied in isolation, and little attention is paid to the direct and indirect effects of climate variability on armed conflict in the same region (Helman et al., 2020). Helman et al. (2020) have shown that multiple causal effects are in play when predicting the risk of armed conflict from climate variability in Africa and the Middle East. Using structural equation modeling (SEM), they quantified direct and indirect effects of climate variables on conflict risk. While such analysis was focused on Africa and the Middle East, areas in Asia that are politically fragile and constantly suffer conflict remain largely unnoticed (Adams et al., 2018). According to the Geo-referenced Event Dataset (GED) (version 20.1) of the Uppsala Conflict Data Program’s (UCDP) database statistics, the number of armed conflict events in South Asia has been increasing from 1850 in 2000 to 2846 in 2015, which accounts for 40% of the armed conflict events in Asia. Besides, South Asia is a less developed part of Asia and has a dense population and a substantial extent of dependence on agriculture (von Uexkull et al., 2016; Wischnath and Buhaug, 2014). However, the effect of climate variability on armed conflict in South Asia is rarely investigated and not well understood.

**Figure 1:**
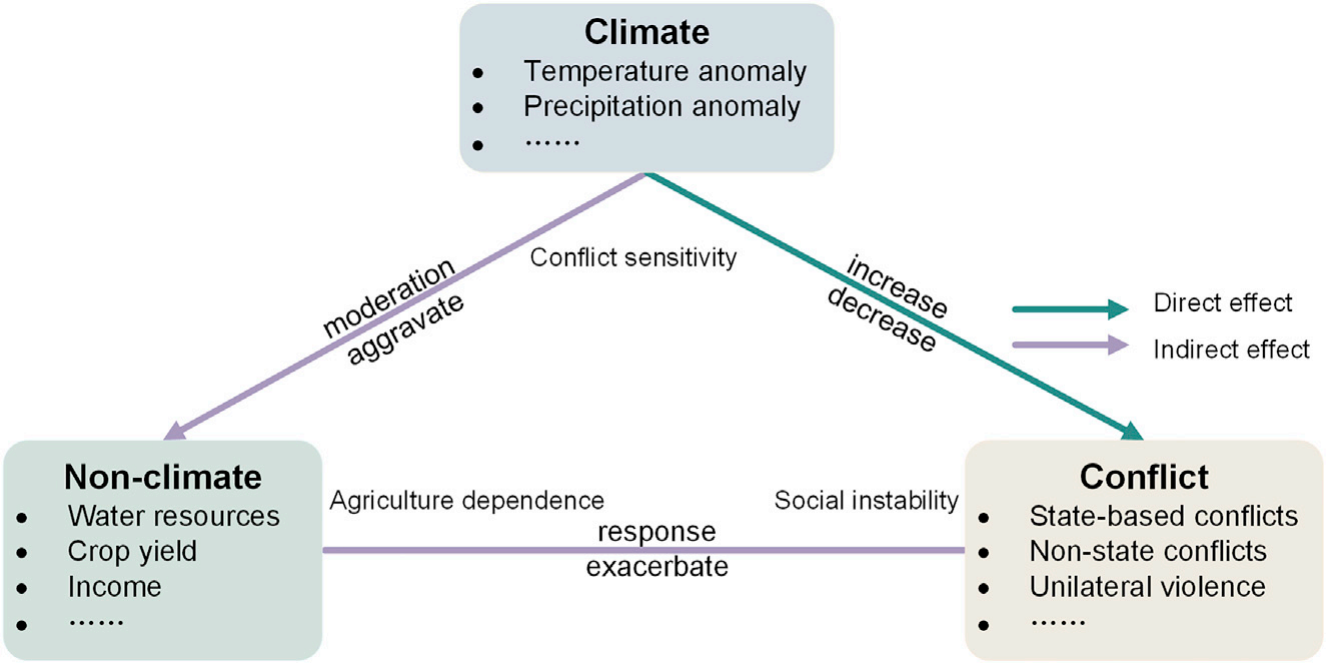
The conceptual framework for the direct (green) and indirect (purple) impacts of climate on conflict

The complicated impacts of climate on armed conflict comprise various factors. Under the conceptual framework of the climate-conflict pathways (Figure 1) (Scheffran et al., 2012), this study seeks to uncover the effects of climate variability on armed conflict in South Asia by simultaneously considering the direct and indirect impacts. The fundamental idea is that climate variability’s impact on armed conflict is both direct and indirect, depending on the changes in non-climate variables such as water security, yield, and income. We assembled an extensive database of armed conflict events and detailed climate and non-climate data (Table S1) covering 2000–2015. SEM is applied to capture underlying linkages of climate variability to armed conflict for its advantages in enabling the exploration of not only the direct and indirect effects simultaneously but also the pathways through which the indirect impacts might manifest. This study aims to develop a theory to understand the complex effects of climate variability on armed conflict in South Asia. This includes Sri Lanka, Nepal, Bhutan, Maldives, Pakistan, Afghanistan, and India as the analyzed countries.

## Results

Figure S1 depicts the spatial distribution of armed conflict events from 2000 to 2015 in South Asia. It shows that most of the events occurred in Afghanistan, Sri Lanka, Nepal, Pakistan-Afghanistan border, and the seven Northeastern states of India (Arunachal Pradesh, Assam, Meghalaya, Manipur, Mizoram, Nagaland, and Tripura). The number of events decreased from 2010 to 2015 after a significant increase since the beginning of the century. The annual average number of armed conflict events in this region was 2662 (Figure S2). Our statistical analysis shows that the numbers of grid-cells with a significant precipitation anomaly were above average (Figures S3 and S4, method details). Grid-cells with a temperature anomaly had an opposite sign to the crop yield anomaly in the same cells (Figure S5, method details). These findings indicate that there may be a link between climatic, non-climatic elements, and armed conflict, while their specific quantitative relationship and causal direction need to be further explored and quantified.

**Figure 2:**
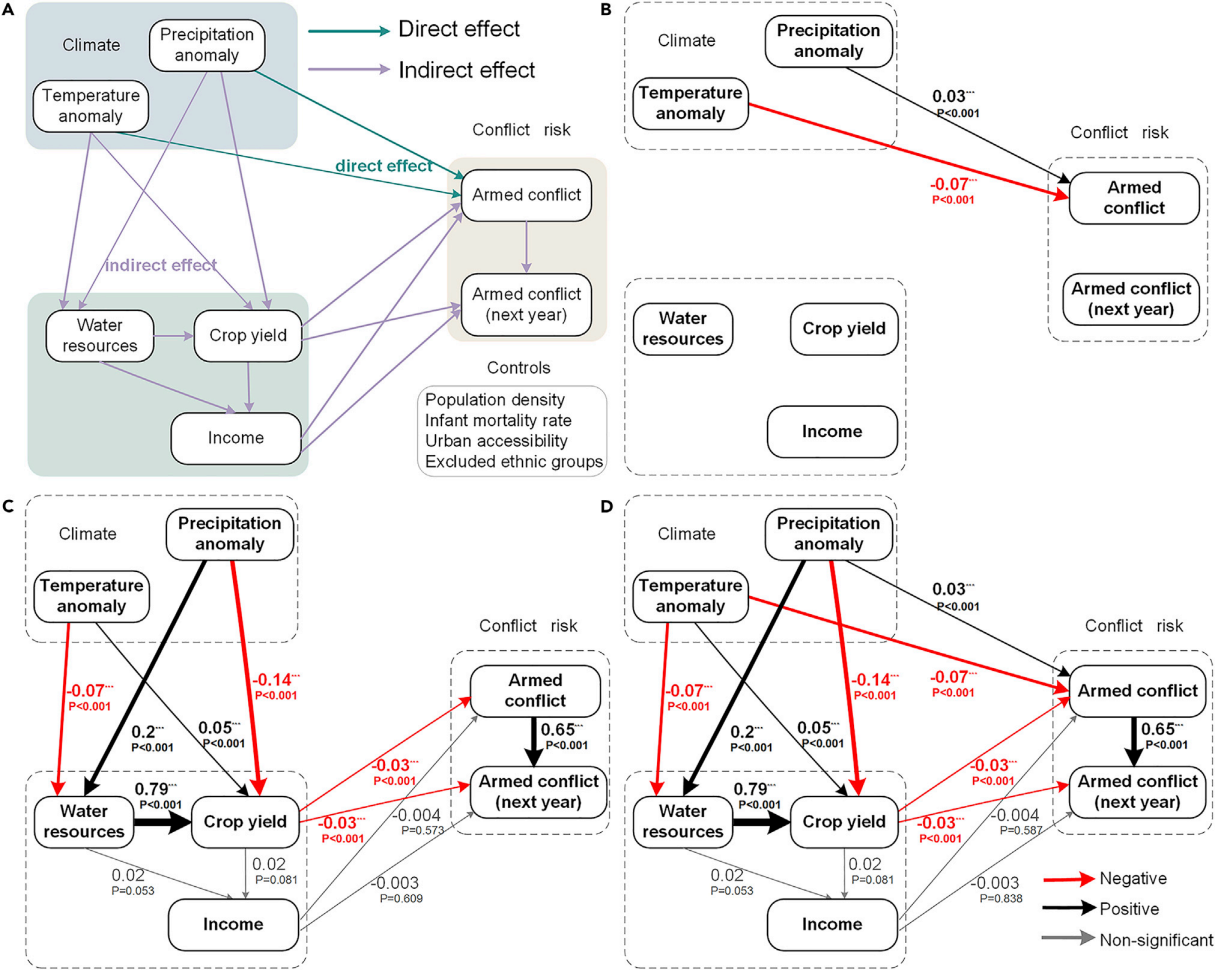
Structural Equation Model showing standardized coefficients of each factor effect on armed conflict (A) The conceptual model. (B) The direct impact of climate variability on armed conflict. (C) The indirect impact of climate variability on armed conflict. (D) The combined impact (direct and indirect) of climate variability on armed conflict. The number next to the arrows indicates the direct effect of standardization, the red arrows indicate a negative effect, the black arrows indicate a positive effect, and the gray arrows are not statistically significant (p > 0.05), while width indicates its importance in the model.

With the help of SEM, multiple direct and indirect effects of climate variability on armed conflict can be quantitatively described (Table 1 and Figure 2). Tests of the SEM model robustness are presented in (method details, Tables S2–S4). Though both precipitation and temperature have significant direct impacts on armed conflict (Figure 2B), precipitation has a positive (0.03) and significant (p < 0.001) effect on armed conflict, while temperature has a negative effect (−0.07). The results also indicate that crop yield has a significant inverse impact on armed conflict in both the present (−0.03) and the following year (−0.03). Likewise, income negatively affects armed conflict in the present and the next year with statistical non-significance (p > 0.05). It is worth noting that the direct effect of current armed conflict has a significant positive impact (0.65) on armed conflict of the following year. These findings are supported by the results from the mean statistics (Figure S3) and correlation analysis (Figure S6), which show that rises in precipitation enhance the chance of armed conflict. At the same time, increases in temperature, crop yield, and income reduce the risk of armed conflict.

**Table 1:** Direct, indirect, and total standardized effects of climate and non-climate variables on armed conflict. NS: not significant, p > 0.05; ∗p < 0.05; ∗∗p < 0.01; ∗∗∗p < 0.001

Predictor	Direct		Indirect		Total	
	Estimate	Standard Errors	Estimate	Standard Errors	Estimate	Standard Errors
Temperature anomaly	−0.068^∗∗∗^	0.006	NS		−0.068^∗∗∗^	0.006
Precipitation anomaly	0.031^∗∗∗^	0.006	−0.001^∗^	0.0001	0.031^∗∗∗^	0.006
Water resources	–		−0.024^∗∗∗^	0.006	−0.024^∗∗∗^	0.006
Crop yield	−0.031^∗∗∗^	0.008	NS		−0.031^∗∗∗^	0.008
Income	NS		–		NS	
Population density	−0.016^∗^	0.009	–		−0.016^∗^	0.009
Infant mortality rate	NS		–		NS	
Urban accessibility	0.054^∗∗∗^	0.006	–		0.054^∗∗∗^	0.006
Excluded ethnic groups	0.063^∗∗∗^	0.006	–		0.063^∗∗∗^	0.006

SEM provides an explicit and detailed path toward understanding the indirect impacts of climate variability on armed conflict (Figure 2C and Table 1). Generally, precipitation has an indirect effect in two ways: (i) (+) precipitation→ (−) crop yield→ (+) armed conflict; (ii) (+) precipitation→ (+) water resources→ (+) crop yield→ (−) armed conflict, where (+) indicates a positive effect and (−) represents a negative effect. The observed indirect effects of precipitation (Figure 2C) canceled each other, resulting in a weak net impact on armed conflict (−0.001). The indirect effect of the temperature on armed conflict was not significant (p > 0.05). Yet, a detailed decomposition provides a complex, contrasting effect which involves a combination of paths: (+) temperature→ (+) crop yield→ (−) armed conflict; (+) temperature→ (−) water resources→ (−) crop yield→ (+) armed conflict. Or, in short, on the one hand, warming causes increases in crop yield, which reduces armed conflict, while, on the other hand, it reduces water resources, which in turn decreases food production that increases armed conflict. Despite their strong effects, these two pathways cancel each other, resulting in the negligible indirect net influence of temperature on armed conflict in South Asia.

## Discussion

Using a large database of armed conflict, and detailed climate and non-climate data, with the SEM, we explore the effects of climate variability on armed conflict in South Asia. Following successful tests of collinearity (Table S5) and reliability (Table S6), we examine the possible direct and indirect effects of climate variability on armed conflict (Figure 2 and Table 1). Our results find that precipitation is associated with increases in armed conflict, which is consistent with some empirical literature that suggested links between plentiful precipitation and armed conflict (Hsiang et al., 2013; Roche et al., 2020; Salehyan and Hendrix, 2014). For instance, it has been reported that Hindu Muslim riots in India are more likely to occur under extreme precipitation conditions (Bohlken and Sergenti, 2010). However, our result indicates that heat may suppress armed conflict, suggesting that the negative affect escape (NAE) might be the primary mechanism in South Asia. According to the NAE model, when rising temperature causes discomfort, people are more interested in finding an escape from the heat than provoking conflict (Bell and Baron, 1976). This finding is similar to previous work on Asia that described a negative effect of temperature on civil war (Wischnath and Buhaug, 2014), contrary to some studies that reported a positive effect of temperature on armed conflict in Africa and across the globe (Carleton and Hsiang, 2016; Helman et al., 2020). We may attribute this discrepancy to the difference in regional climate and geographical environment that shapes social vulnerability, leading to the inconsistent responses of armed conflict to climate variability in South Asia and other regions (Buhaug and von Uexkull, 2021; Guedes et al., 2016).

Previous literature illustrated that under unfavorable weather conditions substantially reducing crop yields and causing huge economic losses, violence might become an alternative source of income for those who can no longer make their living relying on rain-fed agriculture, food supply, or livelihood security (Roche et al., 2020; von Uexkull et al., 2016). Our results indicate that climate variability indirectly affects armed conflict by affecting water and yield, partly supporting the previous research (Koubi, 2019; Theisen et al., 2013). However, unlike previous studies of the link between climate variability and armed conflict, our study particularly paid attention to exploring the indirect effects of climate variability on armed conflict. It is found that the indirect impact of climate variability on armed conflict is complex for multiple causal effects.

On one hand, precipitation, together with temperature, decreases the chance of armed conflict by influencing changes in water resources and crop yield ((+) precipitation→ (+) water resources→ (+) crop yield→ (−) armed conflict; (+) temperature→ (+) crop yield→ (−) armed conflict). For example, a large amount of precipitation may enrich water resources, while the rising temperature warms up the environment thus resisting temperature limitations in both late winter and early spring. The combined effect of these two linkages meets the requirement for water and temperature necessary for increasing crop yield, thereby avoiding food crises and reducing the possibility of conflict (D'Odorico et al., 2020; Meza and Silva, 2009).

On the other hand, temperature and precipitation may also indirectly increase the likelihood of armed conflict ((+) precipitation→ (−) crop yield→ (+) armed conflict; (+) temperature→ (−) water resources→ (−) crop yield→ (+) armed conflict). During the critical period of crop growth, we found that both the increasing frequency of precipitation and temperature events may negatively affect crop yield. The rising temperature would deprive crops of the necessary water for growth by causing a shortage of river runoff and surface water, while the excessive precipitation might destroy the growth of crops by inducing flood and soil erosion (Chaturvedi et al., 2012; Eastin, 2018; Goswami et al., 2006). All of these will wreak havoc on crop growth, resulting in a sharp drop in crop yields and increasing the likelihood of armed conflict. The Naxalite movement in India and the Maoist insurgency in Nepal are two examples where such a dynamic played out (Christian, 2011).

In this study, we find that although the total indirect effects of temperature on armed conflict were not significant, partitioning these net effects into detailed paths revealed its complexity. The result was a weak net effect of temperature, which comprises two strong indirect effects that cancel each other. This means that only investigating net responses may lead to erroneous conclusions. Our quantitative findings can help reconcile the contradictory results of previous studies on temperature and conflict (Buhaug, 2010; Burke et al., 2009). Many studies in this field establish net impacts of climate variability on armed conflict, disregarding the delineation of the impact channels, which may contain offsetting effects from multiple channels leading to a net-zero effect of the studied factor. We may overlook important information about how climate variability impacts armed conflict if we only analyze net effects, limiting our understanding of results and misleading our interpretations of the combined effects of climate variability on armed conflict. The complex contradictory interactions found in this study may also occur at the country level. For example, in the year 2010, Pakistan was greatly hit by a flood caused by large amounts of precipitation (Figure S5). The flood inundated more than 20% of the country and severely affected 29 districts, leading to a series of problems (Abid et al., 2015; Asgary et al., 2012; Busby et al., 2018). Precipitation severely brought down the yield of major crops such as rice and wheat, thus increasing the chance of armed conflict (Figure S7).

In this study, we used the positive and negative anomalies in a single SEM model to explore its direct and indirect effect mechanisms on armed conflict. To account for potential contradictory paths that might cancel each other, we further constructed four new SEM models, separating our datasets to positive and negative anomalies. Our results using a single SEM model and separate SEM models for positive and negative anomalies are generally consistent (Figure S8), implying that both negative and positive precipitation and temperature anomalies have complex effects on armed conflict.

### Limitations of the study

This research systematically reveals the complex linkages of climate variability effects on armed conflict in South Asia. Future research can be carried out on the following aspects to comprehensively explore this complex impact of climate variability on armed conflict globally. First, although we have sorted out the possible paths to decipher the impacts of climate variability on armed conflict based on previous studies. More complicated paths may exist between them, which can be explored in the future with a study design that can try and incorporate them simultaneously. Second, we are aware that in the nonlinear case the direct and indirect effects of climate variability on armed conflict are more complex than in a simple linear form. Such complex nonlinear effects are difficult to capture when taking multiple variables and pathways into account. We see the challenges of simultaneously considering the nonlinear and indirect effects of climate variability on armed conflict. In future work, we may attempt to provide a comprehensive view of climate variability to nonlinearly describe its direct and indirect impacts on armed conflict. Lastly, our research focuses on South Asia for its distinctive characteristics, including dense population, underdeveloped economy, and a substantial extent of dependence on agriculture as well as frequent armed conflict. However, other regions on this planet may contain different characteristics. It is encouraged to put efforts into researching the effects of climate variability on armed conflict in other parts of the world.

## STAR★Methods

### Key resources table

**Table:** 

REAGENT or RESOURCE	SOURCE	IDENTIFIER
**Software and algorithms**		

IBM SPSS	IBM	https://www.ibm.com/analytics/spss-statistics-software
IBM SPSS AMOS	IBM	https://www.ibm.com/products/structural-equation-modeling-sem

### Resource availability

#### Lead contact

Further information and requests should be directed to the lead contact, Fangyu Ding (dingfy@igsnrr.ac.cn).

#### Materials availability

This study did not generate any new reagents.

### Experimental model and subject details

This study does not include experiments or subjects.

### Method details

#### Data sources

The information of armed conflict data is acquired from the GED (version 20.1) of the UCDP comprehensive data about organized violence (Kreutz, 2010). This dataset covers three types of conflict events (state-based conflicts, non-state conflicts, and unilateral violence) from 1989-2020. It defined an event as an organized actor using armed force against another organized actor or civilians, resulting in at least one direct death at a specific location on a specific date (Kreutz, 2010). We confined our analysis to 2000–2015 since this was the period when all datasets overlapped. We got the armed conflict events of each year through selecting the identifier (Year) in the UCDP GED dataset, then we assigned every UCDP conflict event of each year in South Asia to the grid with a cell size of 0.5° × 0.5°, and the number of armed conflict events per year in each grid is summarized. Since we focused on the impacts of climate variability on conflict, Equation 1 adopted by the previous literature was used to convert the number of events in each grid cell to binary values of 0 and 1, where 1 indicated experienced violence during this year and 0 otherwise (Ge et al., 2022).

(Equation 1) $$\text{Conflict}\;{\text{Risk}}_{ji}=\left\{ \begin{array}{l} 1\;if\;a\;conflict\;event\;occurs\;at\;grid\;i\;in\;year\;j\\ 0\;if\;no\;conflict\;event\;occurs\;at\;grid\;i\;in\;year\;j \end{array}\right.$$

Temperature anomaly is an important indicator of climate variability as well as one factor associated with social conflict (O'Loughlin et al., 2014). We extract the dataset of monthly maximum temperatures from the Climate Hazards Center Infrared Temperature with Stations (CHIRTS) dataset to measure temperature anomalies. CHIRTS can help identify, quantify, and explore changes in extreme temperatures while providing valuable resources for analyzing extremes of climate (Helman et al., 2020). To generate annual temperature anomaly data from 2000 to 2015 with a spatial resolution of 0.5°, we first calculate the annual level of maximum temperature for each cell. After that, we convert the yearly temperature anomaly data covering the period from 2000 to 2015 by z-score processing (deviations from the annual average maximum temperature 1983-1999, (Equation 2)). Finally, we re-sample annual temperature anomaly data to a spatial resolution of 0.5.

(Equation 2) $$T_{ij*}=\frac{T_{ij}-\mu_{Ti}}{\delta_{Ti}}$$

Where $T_{ij*}$ is temperature anomaly of grid i in year *j*, $T_{ij}$ is the annual maximum temperature of grid *i* in year *j*, $\mu_{Ti}$ is the annual average maximum temperature of grid *i* covering the period 1983–1999, and $\delta_{Ti}$ is the standard deviation of the annual maximum temperature of grid *i* covering the period 1983–1999.

Precipitation anomaly is one of the reliable indicators of climate variability. It was also found related to the conflict in previous studies (Hsiang et al., 2013). We derive a monthly precipitation dataset from the Climate Hazards Group Infrared Precipitation with Stations (CHIRPS) to measure precipitation anomalies (Mack et al., 2021). CHIRPS provides instant and reliable precipitation information on a global scale, which helps monitor agricultural drought and environmental changes (Shen et al., 2020). To generate annual precipitation anomaly data from 2000 to 2015 with a spatial resolution of 0.5°, we convert the monthly precipitation dataset into yearly precipitation anomaly data covering the period from 2000 to 2015 by z-score processing (deviations from the annual average precipitation 1983–1999, Eqution 3). We then aggregate the original resolution into a spatial resolution of 0.5°.

(Equation 3) $$P_{ij*}=\frac{P_{ij}-\mu_{Pi}}{\delta_{Pi}}$$

Where $P_{ij*}\;$ is precipitation anomaly of grid i in year j, $P_{ij}$ is the annual precipitation of grid i in year j, $\mu_{Pi}$ is the annual average precipitation of grid i covering the period 1981–1999 and $\delta_{Pi}$ is the standard deviation of annual precipitation of grid i coving the period 1981–1999.

Regarding the non-climate variables, we determine crop yields, income, and water resources to be indicators that influence the possibility of armed conflict in South Asia. Our choice of variables was grounded on the fact that the heavy agricultural dependence of this region makes its agricultural and national incomes prone to fluctuating crop yields, which itself is sensitive to climate variation and water resource distribution (Adams et al., 2018). In addition, what usually comes after crop losses would be the government capacity diminished by the reduced tax revenue resulting from falling incomes, which would soon be reflected in income (Koubi, 2019). Also, one of the most significant issues in South Asia that may not be missed is water scarcity that has aroused social grievance and exacerbated the already dire economic situation (Vinke et al., 2017). Therefore, we chose these three variables to represent the mediators of climate variability affecting armed conflict risk.

Climate variability exacerbates water shortages, which affect agricultural production, and in turn, increases the risk of conflict making water resources an important mediator between climate variability and conflict (Ikhuoso et al., 2020). We use the soil moisture data as a surrogate for water resources, which plays a critical role in estimating soil water evaporative fluxes, drainage, and runoff, providing great potential for continental water resource assessment (Abowarda et al., 2021).

Climate variability has adverse effects on crop yields that increase the incentives for conflict (Kelley et al., 2015). We use the normalized difference vegetation index (NDVI) in this study as a substitutive indicator for crop yield (Zheng et al., 2019). NDVI can both estimate crop-growing conditions directly and assess the climate variability influence on crops, which improves the crop yield prediction (Fensholt and Proud, 2012).

With the negative impact of climate variability on water resources and agriculture, the spread of poverty may increase grievances that create conditions for conflict (von Uexkull et al., 2016). Although the income and welfare statistics are currently unavailable, we refer to the conflict studies of von Uexkull et al. (2016) and use the global nighttime light composite data from the Visible Infrared Imaging Radiometer Suite (VIIRS) day-night band (DNB) carried by the Suomi National Polar orbiting Partnership (NPP) satellite to estimate the income states and dynamics (Levin and Zhang, 2017; Shi et al., 2014). Lights at night have been shown to be a good proxy for income and are considered to correlate well with GDP, poverty, and other socioeconomic welfare variables (Chen and Nordhaus, 2011; Henderson et al., 2011).

Previous research suggested that in addition to variables related to climate, the factors that represent politics, economy, and society are important to study conflict (Fearon and Laitin, 2003; O'Loughlin et al., 2012; von Uexkull et al., 2016). We refer to previous studies while considering the availability of data, using the following data to represent politics, economy, and society factors that affect conflict.

Conflict is more likely to occur in areas where ethnic groups are marginalized (Cederman et al., 2010). To estimate the political dimension of vulnerability, we include a binary exclusion variable that identifies whether the group is excluded from executive state power according to the EPR-ETH dataset (Vogt et al., 2015).

As closeness to large cities may increase the ease of information dissemination, these locations can be attractive targets to the insurgents during certain conflicts (Fearon and Laitin, 2003). We use the time required for individuals to reach their most accessible city to capture spatial variations in the ease of disseminating a message (Weiss et al., 2018).

Given that violent conflict is a form of collective violence, the regions with high population density are usually close to major cities and rich in natural resources, rendering them high-risk targets to be attacked by the rebels (Raleigh and Hegre, 2009). Therefore, we consider population density derived from the Gridded Population of the World (GPW v4.10) created by the Center for International Earth Science Information Network and Socio-Economic Data and Applications Center of Columbia University (Center for International Earth Science Information Network, 2011) as a driver of conflict risk.

Socioeconomic status is another important determinant of conflict risk (Collier and Hoeffler, 2004). We use infant mortality rate from the Global Subnational Infant Mortality Rates, Version1 (GSIMR.v1) (Interagency Group for Child Mortality Estimation, 2011) as a proxy of socioeconomic status because the infant mortality rate can also serve as a broad measure of socioeconomic status when measures like gross domestic product per capita are difficult to obtain on grid-scale (Interagency Group for Child Mortality Estimation, 2011; O'Loughlin et al., 2012).

#### Structural equation modeling

We used the SEM to fit data because it enables us to both propose hypotheses about how each variable works (correlations, direct, and indirect relationships among variables) and to test these relationships with real data (Keller et al., 1998). Therefore, SEM is particularly suitable for confirming the direct and indirect effects of climate and non-climate factors on armed conflict (Adedeji et al., 2016).

Previous studies showed that the risk of conflict might be greater in places where conflict already occurred (Schutte and Weidmann, 2011), and the indirect effect (through crop yields, water resources, and income) of climate anomaly on armed conflict may have a certain time-lag, so we included one-year time lag of conflict events to control these relations.

To account for the differences in the effects of politics, economy, and social factors on armed conflict while considering the availability of data at both spatial and temporal scales, we included the following set of control variables that have been associated with conflict in previous conflict studies research in SEM: exclusion of political groups, population density, infant mortality rate and urban accessibility (Fearon and Laitin, 2003; O'Loughlin et al., 2012; von Uexkull et al., 2016).

We developed an SEM model (Figure 2A) with control variables and time-lag structure to estimate the direct and indirect effect of climate variability on armed conflict. In this model, we hypothesized that climate variability has not only direct effects on current armed conflict but also indirect effects on both current and next year's armed conflict through crop yields, water resources, and income. The SEM model was run in IBM SPSS AMOS, and a bootstrap procedure (2000 replications) estimated the standard errors of the coefficients for the final model at the 95% confidence interval.

#### Assessment of goodness fit

We defined the following indices to evaluate the goodness of fitting the SEM model (Bentler and Bonett, 1980; Pollman, 2014). The comparative fit index (CFI), which indicates the improvement proportion of a hypothesized model relative to a baseline model, was set to be an acceptable fit if > 0.9. According to the goodness-of-fit index (GFI), values close to 0.9 indicated a good fit. The adjusted goodness-of-fit index (AGFI), which was adjusted for degrees of freedom, indicated a good fit if > 0.8. The root-mean-square error of approximation (RMSEA) assessed how far a hypothesized model is from a perfect model, where a value of 0.1 or less was considered a mediocre fit. The standardized root means square residual (SRMR) evaluates the overall fit of the model, with values less than 0.1 indicating an acceptable fit. The goodness fit statistics results suggest that all indexes performed well except for the mediocre fit of RMSEA, meaning that the model functions well in describing the data (Table S6).

#### Robustness check

To check how sensitive the results of SEM modeling are to the time lag structure and control variables, we constructed another three separate SEM models. When we drop the time lag structure, the result for the effect of climate variability on armed conflict exhibits little change (Table S2). Similarly, we drop all control variables to little effect (Table S3). As a matter of fact, the SEM yields largely similar results when we drop the time lag structure and all control variables at the same time (Table S4). These results indicate that the effects of climate variability on armed conflict are independent of control variables and time lag structure as we have measured them.

#### Explanation of SEM results

In this study, we used the relative anomaly for the deviations from normal, including positive and negative deviations with opposite numerical signs. For a positive deviation, a larger value means a larger deviation from the multi-annual mean (zero, in our case). However, for a negative deviation, a larger value means a smaller deviation from the multi-annual mean. Taking the effect of precipitation on water resources as an example, we can see that for a negative precipitation anomaly (precipitation below the mean), water resources decrease as the negative deviation become larger (Figure S9). In contrast, water resources increase with the rising positive precipitation anomaly (precipitation above the mean, i.e., larger + P →larger WR and larger –P →less WR).

### Quantification and statistical analysis

Since the multi-collinearity of the data may affect the estimation of SEM, we use the tolerance and variance inflation factor (VIF) in IBM SPSS to test the collinearity of the data (Bagozzi and Yi, 2012). When VIF<10 and tolerance >0.1, it indicates that there is no collinearity (Snee, 1977). The result shows that the VIF value between the data is less than 10 and the tolerance is greater than 0.1 (Table S5), which means that the collinearity of the data in this study is within the acceptable range of SEM.

## Data Availability

Data: All data reported in this paper will be shared by the lead contact upon request. Code: This paper does not report original code. Any additional information required to reanalyze the data reported in this paper is available from the lead contact upon request.
