# Supplementary material for: Exploring the direct and indirect impacts of climate variability on armed conflict in South Asia
Source: iScience. 2022 Oct 19;25(11):105258. doi: 10.1016/j.isci.2022.105258 (PMC9684034; doi:10.1016/j.isci.2022.105258)
Supplement: Document S1. Figures S1–S9 and Tables S1–S6 [file mmc1.pdf]

iScience, Volume *25*

Supplemental information

Exploring the direct and indirect impacts of climate variability on armed conflict in South Asia

Xiaolan Xie, Mengmeng Hao, Fangyu Ding, David Helman, Jürgen Scheffran, Qian Wang, Quansheng Ge, and Dong Jiang

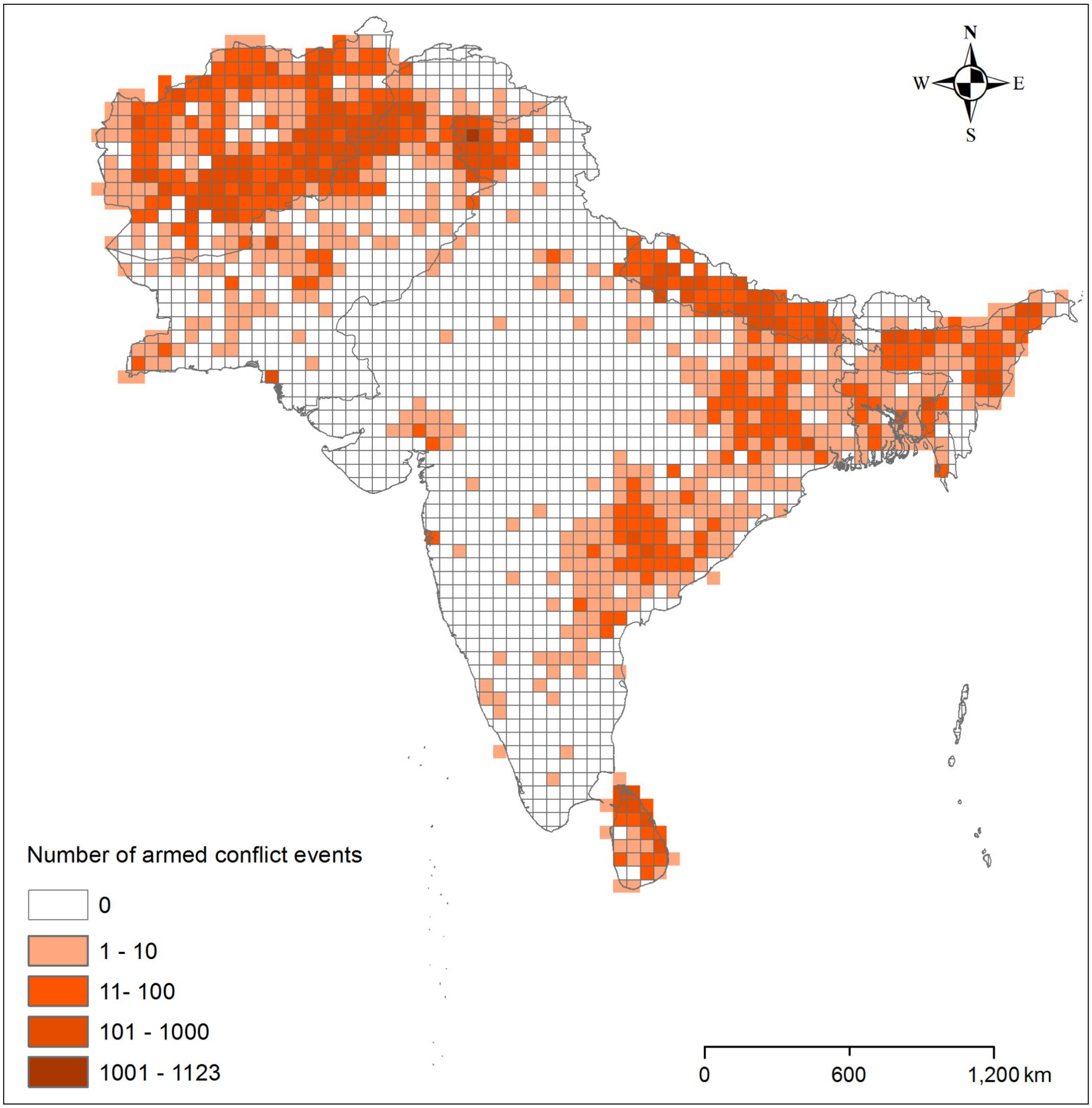

Figure S1. Spatial distribution of armed conflict events for 2000-2015 in South Asia, related to Results.

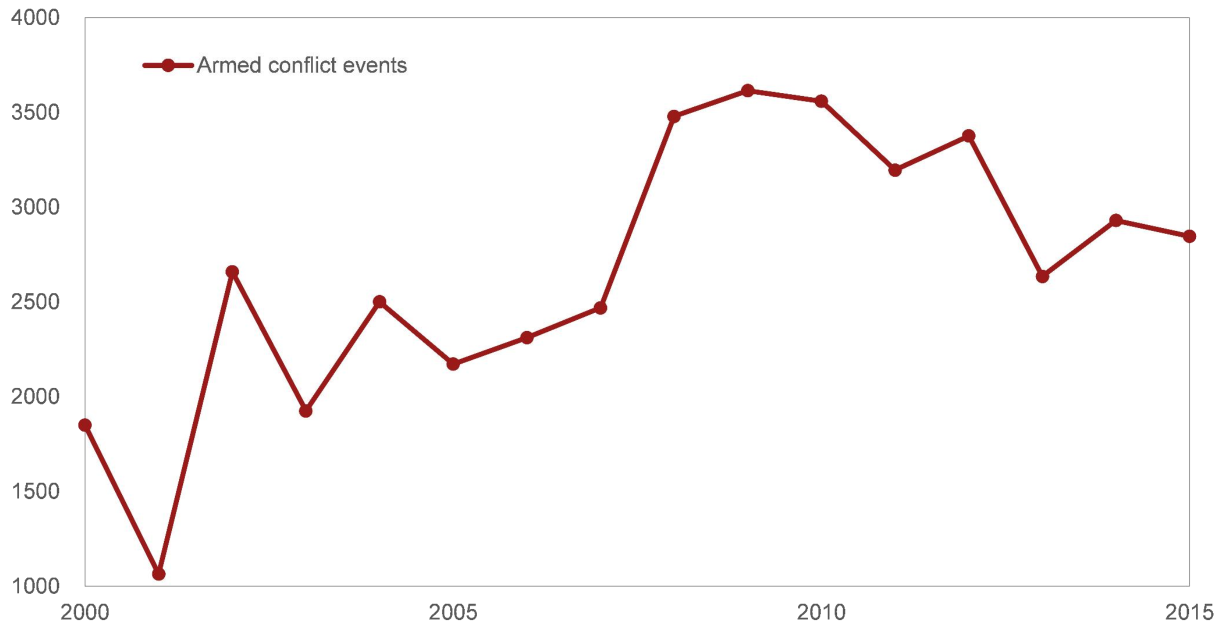

Figure S2. Statistics of armed conflict events for 2000-2015 in South Asia, related to Results.

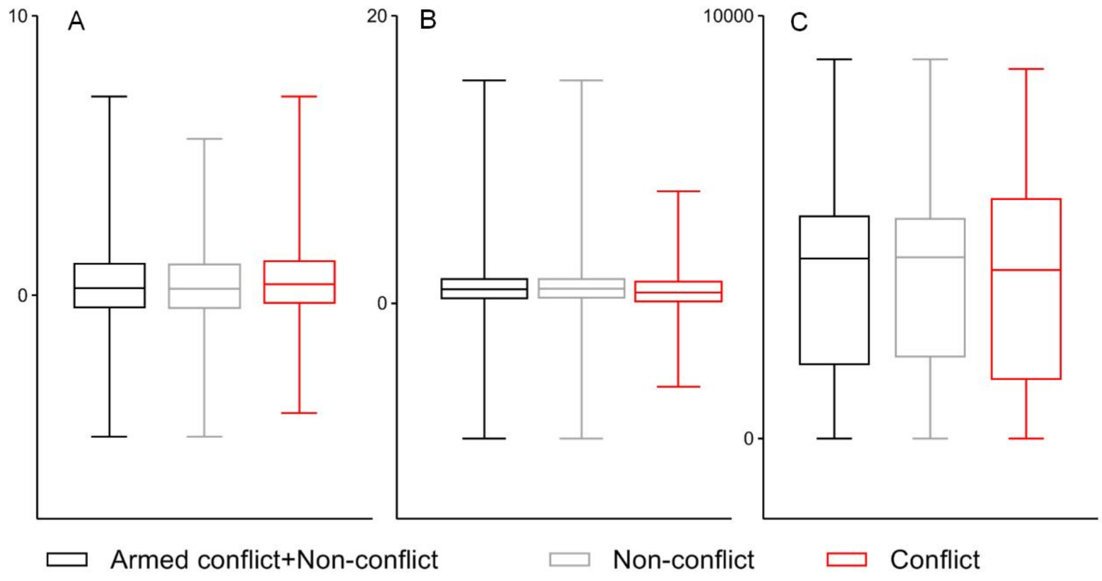

Figure S3. Boxplots, related to Results. Showing median, 1st and 3rd quantiles, upper and lower bounds of (A) mean annual precipitation anomaly, (B) mean annual temperature anomaly, (C) mean crop yield for conflict (red), non-conflict (gray), and all (conflict+non-conflict) (black) grids.

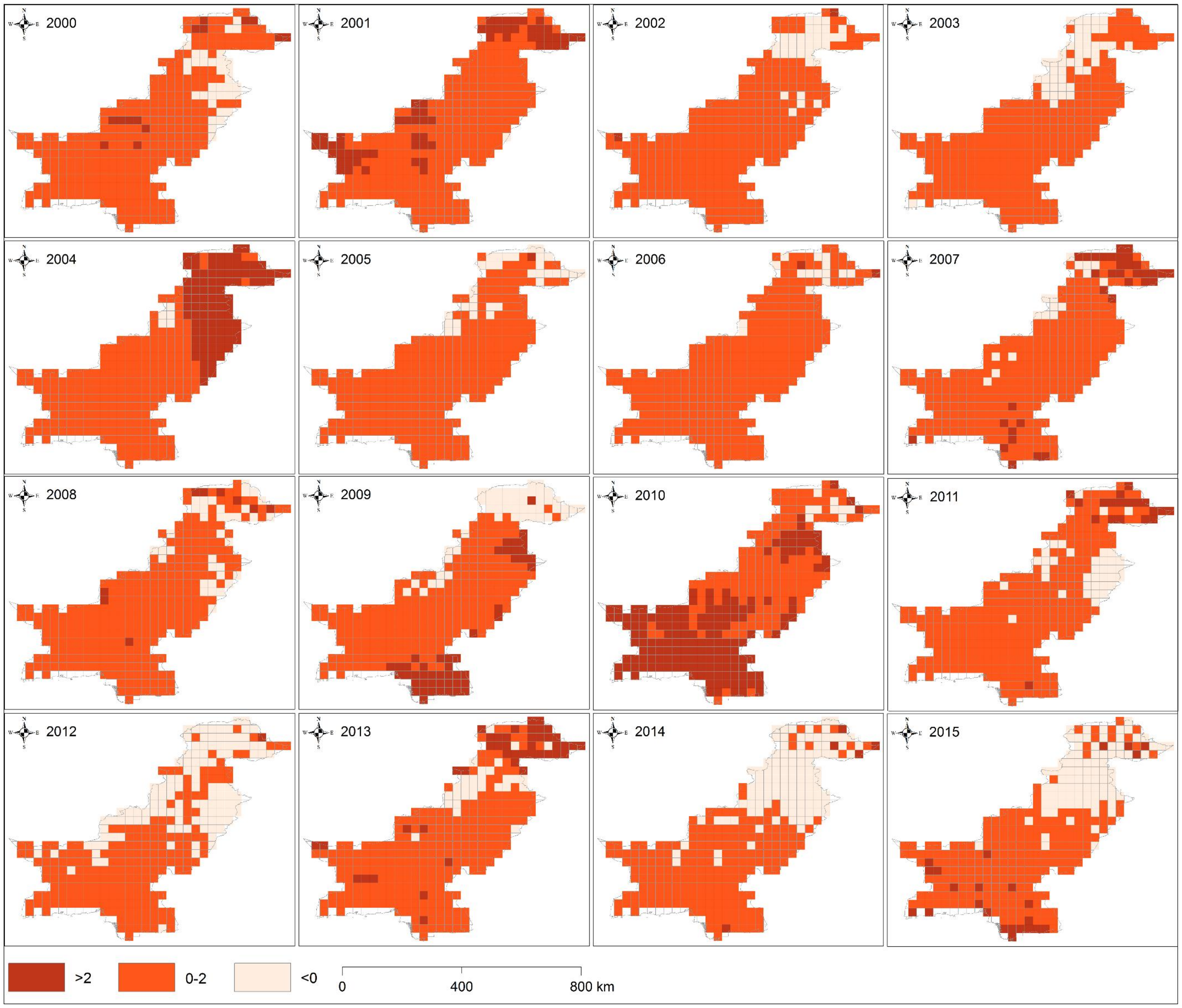

Figure S4. Temperature anomaly in Pakistan from 2000 to 2015, related to Results. The value of grids less than 0 indicates a negative temperature deviation and greater than 0 indicates a positive temperature deviation.

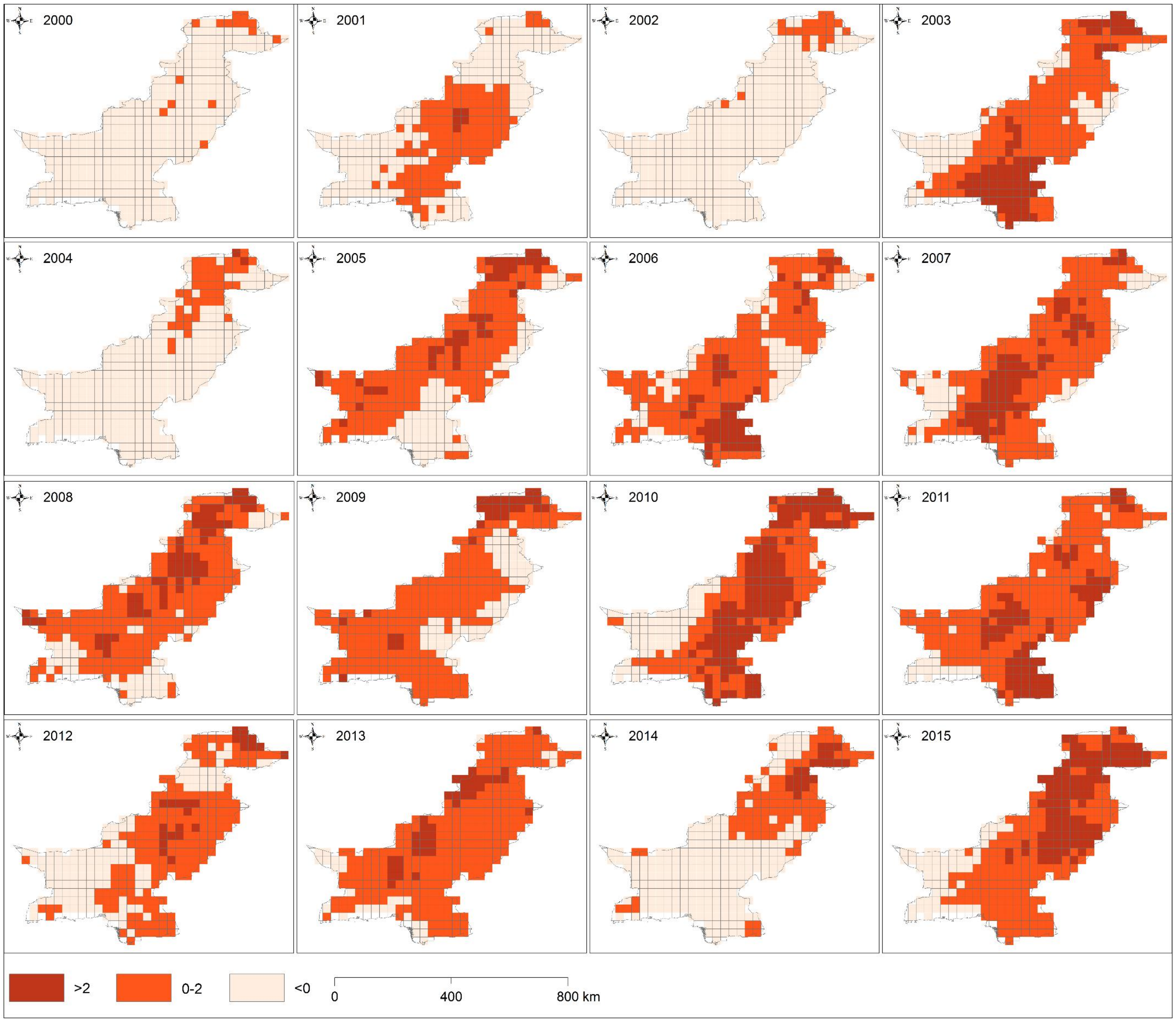

Figure S5. Precipitation anomaly in Pakistan from 2000 to 2015, related to Results. The value of grids less than 0 indicates a negative precipitation deviation and greater than 0 indicates a positive precipitation deviation.

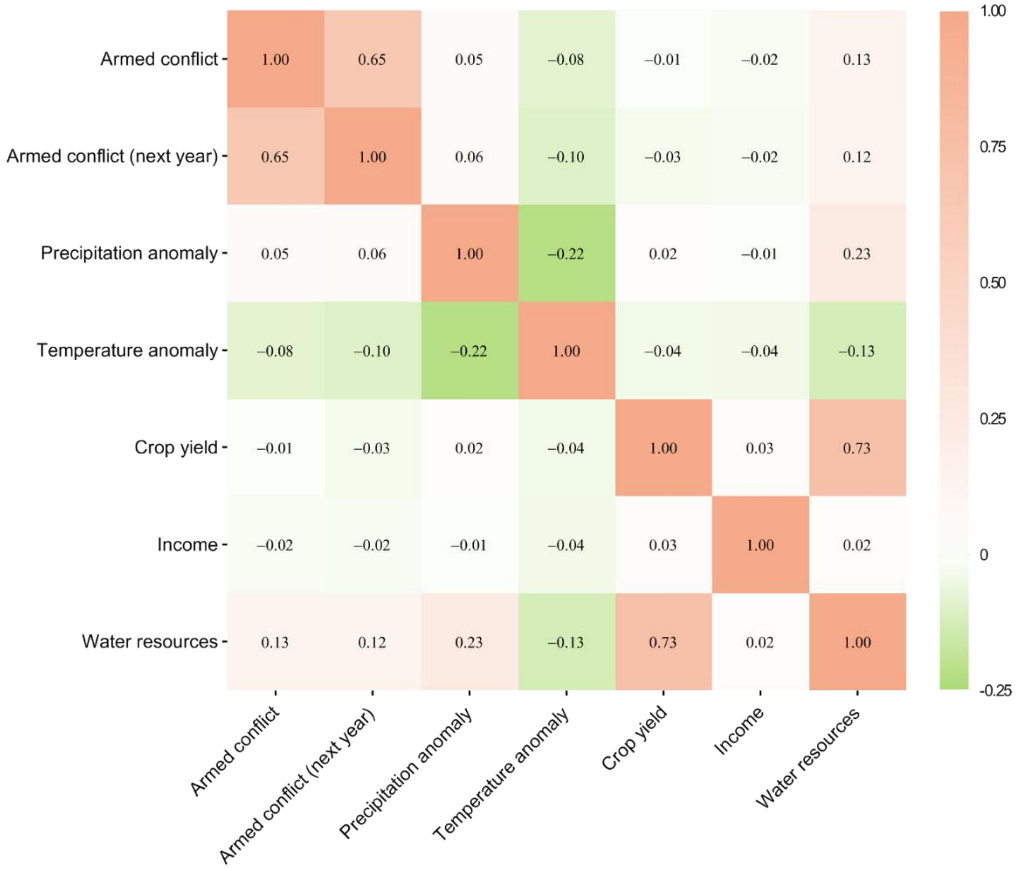

Figure S6. Correlation coefficient matrix of all variables, related to Results.

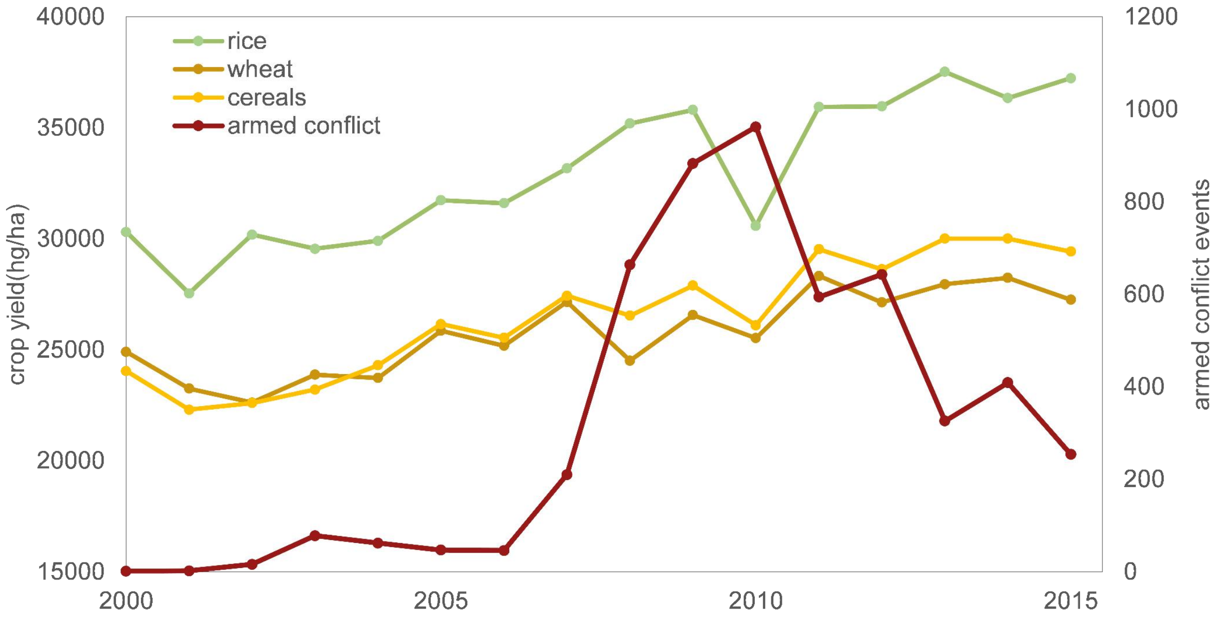

Figure S7. Statistics of crop yield and armed conflict events from 2000 to 2015 in Pakistan, related to Discussion.

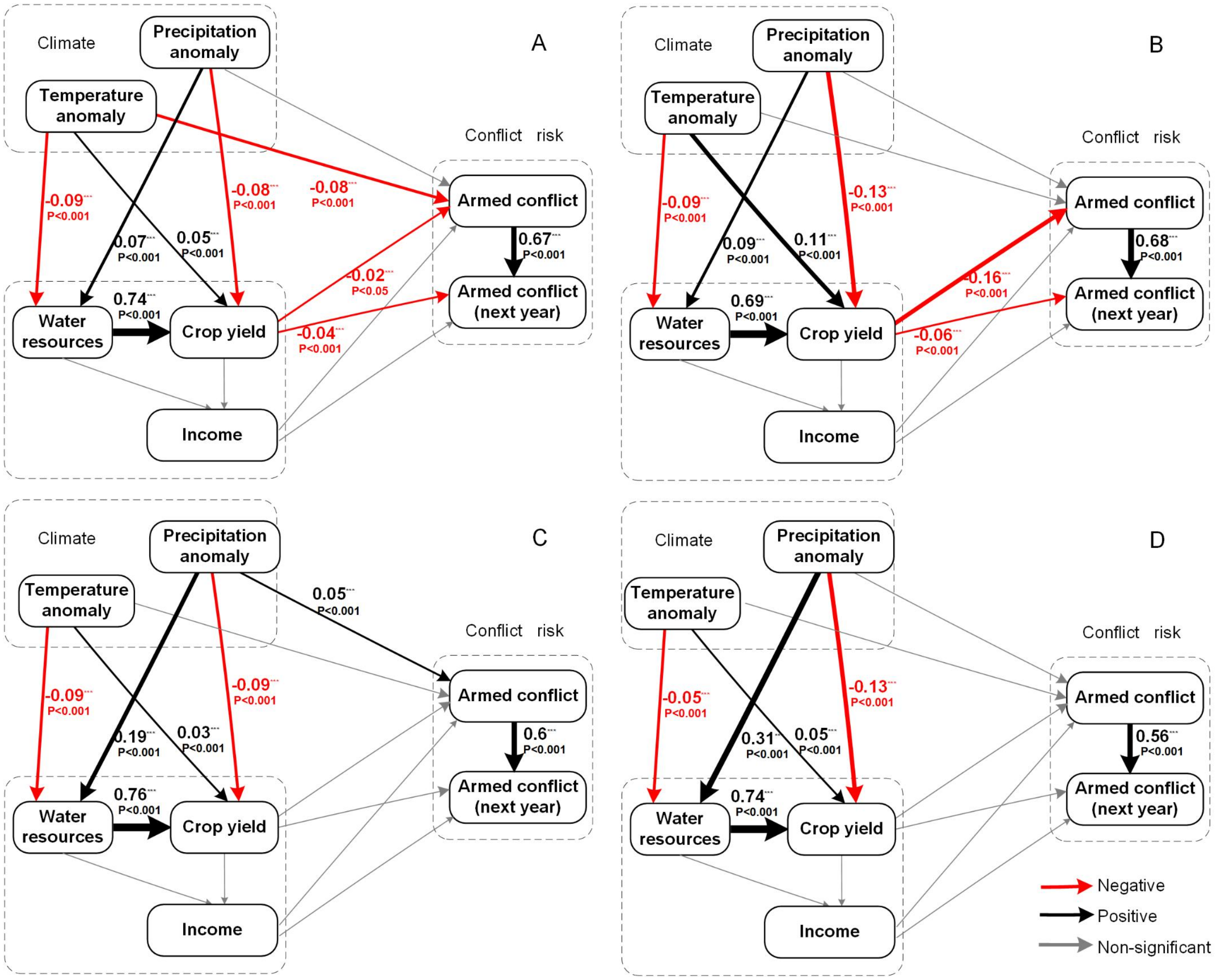

Figure S8. Structural Equation Model showing standardized coefficients of each factor effect on armed conflict, related to Discussion. (A) positive precipitation deviation and positive temperature deviation, (B) positive precipitation deviation and negative temperature deviation, (C) negative precipitation deviation and positive temperature deviation, and (D) negative precipitation deviation and negative temperature deviation. The number next to the arrows indicates the direct effect of standardization, the red arrows indicate a negative effect, the black arrows indicate a positive effect, and the gray arrows are not statistically significant (P>0.05), while width indicates its importance in the model.

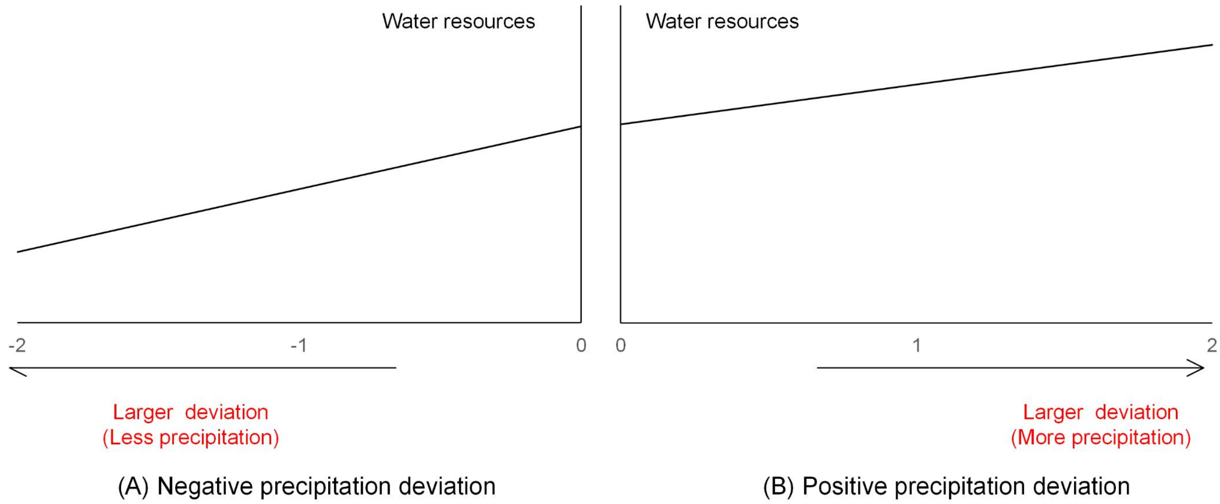

Figure S9. A diagram of the effect of (A) negative precipitation deviation and (B) positive precipitation deviation on water resources, related to STAR Methods.

Table S1. List of datasets, variables used, and research center where the dataset was produced, and the spatial resolutions of each dataset, related to STAR Methods.

Indicators	Dataset	Variable	Temporal resolution	Spatial resolution
Conflict	UCDP GED.v20.1	Armed conflict	Year	Single villages
Climatic	CHIRTS	Temperature [max]	Month	0.05°
	CHIRPS.v2	Precipitation	Month	0.05°
Non-climatic	Noah-MP simulation	Soil moisture	Year	0.1°
	GIMMS	NDVI	Year	10 km
	NPP	Night-light	Year	500 m
	GeoEPR	Ethnic Power Relations	Year	Cell
	GPW v4	Population density	Year	Cell
	SEDAC	Infant mortality rate	Year	Cell
	-	Urban accessibility	Year	Cell

Table S2. Standardized effects of climate and non-climate variables on armed conflict (no time lag structure), related to STAR Methods. NS: not significant, P>0.05; $^{*}P < 0.05$; $^{**}P < 0.01$; $^{***}P < 0.001$

Predictor	Direct		Indirect		Total	
	Estimate	Standard Errors	Estimate	Standard Errors	Estimate	Standard Errors
Temperature anomaly	-0.068***	0.006	NS		-0.068***	0.006
Precipitation anomaly	0.031***	0.006	-0.001*	0.0001	0.037***	0.006
Water resources	-		-.016***	0.006	-.016***	0.006
Crop yield	-0.03*	0.008	NS		-0.019***	0.008
Income	NS		-		NS	
Population density	-0.016*	0.009	-		-0.016*	0.009
Infant mortality rate	NS		-		NS	
Urban accessibility	0.055***	0.006	-		0.055***	0.006
Excluded ethnic groups	0.065***	0.006	-		0.065***	0.006

Table S3. Standardized effects of climate and non-climate variables on armed conflict (no control variables), related to STAR Methods. NS: not significant, P>0.05; *P < 0.05; **P < 0.01; ***P < 0.001

Predictor	Direct		Indirect		Total	
	Estimate	Standard Errors	Estimate	Standard Errors	Estimate	Standard Errors
Temperature anomaly	-0.064***	0.006	NS		-0.064***	0.006
Precipitation anomaly	0.030***	0.006	-0.001*	0.0001	0.03***	0.006
Water resources	-		-.022***	0.005	-0.022***	0.005
Crop yield	-0.028***	0.007	NS		-0.028***	0.007
Income	NS		-		NS	

Table S4. Standardized effects of climate and non-climate variables on armed conflict (no control variables and time lag structure), related to STAR Methods. NS: not significant, P>0.05; *P < 0.05; **P < 0.01; ***P < 0.001

Predictor	Direct		Indirect		Total	
	Estimate	Standard Errors	Estimate	Standard Errors	Estimate	Standard Errors
Temperature anomaly	-0.067***	0.006	NS		-0.067***	0.006
Precipitation anomaly	0.032***	0.006	-0.003*	0.0001	0.029***	0.006
Water resources	-		-0.021***	0.005	-0.021***	0.005
Crop yield	-0.027***	0.007	NS		-0.027***	0.007
Income	NS		-		NS	

Table S5. Collinearity statistics in the data, related to STAR Methods. VIF<10 and tolerance >0.1, which indicates that there is no collinearity in the used data.

Variable	VIF	Tolerance
Precipitation anomaly	1.146	0.872
Temperature anomaly	1.055	0.948
Water resources	2.473	0.404
Crop yield	2.739	0.365
Income	1.062	0.942
Exclusion of political groups	1.291	0.775
Population density	1.11	0.901
Infant mortality rate	1.449	0.69
Urban accessibility	1.198	0.834

Table S6. Fit indexes (CFI, GFI, AGFI, RMSEA and SRMR) for the SEM model, related to STAR Methods.

Index	value	statistical conditions
CFI	0.903	≥0.9
GFI	0.931	≥0.9
AGFI	0.802	≥0.8
RMSEA	0.118	< 0.1
SRMR	0.096	<0.1
